# Physical and mental health status and influencing factors of older adults in community-dwelling in Northeast China: a cross-sectional study

**DOI:** 10.3389/fpubh.2024.1465937

**Published:** 2024-11-21

**Authors:** Mingcheng Gao, Ziyang Chen, Jing Li, Yue Liu, Run Lv, Huanhong Chen, Lulu Zhang, Yining He, Na Guo, Yuping Zhang, Ying Zhang, Xin Dai

**Affiliations:** ^1^School of Public Health, Dalian Medical University, Dalian, China; ^2^Yunkang School of Medicine and Health, Nanfang College, Guangzhou, China; ^3^Department of Development & Planning, Dalian Medical University, Dalian, China

**Keywords:** influencing factors, physical and mental health, community, older adults, China

## Abstract

**Background:**

Between 2020 and 2022, the COVID-19 pandemic spread globally, and the implementation of preventive measures led to reduced outdoor activities for older adults, resulting in a decline in social functioning. This study aims to improve community-based health interventions tailored to older adults experiencing physical and psychological declines following the COVID-19 pandemic.

**Methods:**

This study utilized previous data mining results to estimate the demand for community health services for older adults. It involves collecting questionnaire responses to understand the basic characteristics and lifestyle behaviors of older adults. The severity of health issues among older adults was assessed using the KCL and GHQ-12 scales. Statistical analyses included descriptive statistics, chi-square tests, *t*-tests, ANOVA, non-parametric tests as applicable, and stratified binary logistic regression to determine the factors influencing the health status of older adults.

**Results:**

Over 60% of the older adult population suffers from chronic diseases, and more than 70% do not participate in social activities. In the overall older adults, the detection rate for poor health is 15.60%. Chronic illness, reduced ability to perform daily activities, anxiety, poor self-rated health, sleep disturbances, and nutritional imbalance were identified as key risk factors affecting the health of older adults in the community.

**Conclusion:**

Older adults mainly engage in physical exercise, maintain a healthy lifestyle, and control their diet as self-care strategies. Early signs of frailty are characterized by declines in lower limb muscle function and memory. The most common manifestation of poor health among older adults is anxiety.

## Background

Data from the seventh national population census of China showed that by the end of 2020, the population aged 65 and older accounted for 13.50% of the total population, an increase of 4.63% from 10 years ago (1). A significant proportion of the older adult population suffers from chronic diseases, cognitive decline, and mental health problems, which subsequently lead to increased social challenges (2, 3). Among these issues, frailty is a matter of particular concern (4). Multiple large prospective cohort studies have demonstrated a clear association between frailty and adverse outcomes, including those focusing on community-dwelling older adults, which identified the worst outcomes among the frailest individuals (5, 6). Frailty is a dynamic process, and its progression often results in worsening disability, increased risks of falls, hospitalization, and mortality. These findings underscore the importance of addressing frailty and the necessity for early intervention measures in the older adult population (7).

To effectively address the health challenges of older adults, “healthy aging” is viewed as a key goal for improving their quality of life. Healthy aging involves creating a supportive social environment, preventing disease, maintaining physical and mental functions, and slowing the decline in physiological, psychological, and social capacities. Through comprehensive intervention measures, healthy aging can help older adults maintain a higher quality of life. However, the global COVID-19 pandemic from 2020 to 2022 and its associated control measures restricted outdoor activities and social opportunities for older adults, leading to a decline in their physical mobility and significant psychological stress. A reduction in social activities can exacerbate loneliness and anxiety among older adults, further diminishing their social functioning.

This research was conducted to effectively implement community-based health interventions for older adults. Liaoning Province has the highest aging rate in China, with 17.42% of its population aged 65 and older (8). This study focuses on older adults living in Dalian communities, Liaoning Province. Through questionnaire surveys, it aims to gain an in-depth understanding of the physical frailty and mental health of independently living older adults. Additionally, the research analyzes key factors affecting frailty and mental health. Considering local community-based health management policies, this study provides insights for developing effective health intervention strategies and management measures.

## Methods

The participants in this study were from seven communities in Dalian. The study was conducted in two phases, with recruitment taking place in 2020 and 2022. With the assistance of community health service centers, we used a convenience sampling method to select older adults who met the inclusion criteria. The primary objective of this study was to establish early interventions for the health capabilities of older adults, focusing on those capable of basic self-care. All participants provided written informed consent before participating in the study.

Based on existing literature and estimates of frailty and mental health issues among older adults in the community, we used G*Power software to calculate the sample size. The study’s statistical power was set at 0.80 with a significance level of 0.05. The minimum required sample size was 1,200 participants to ensure statistical significance. Ultimately, we recruited 1,362 older adults and collected 1,322 valid questionnaires, resulting in an effective response rate of 97.1%. This study used paper questionnaires for data collection. Community health workers distributed paper questionnaires to older adults and assisted them in completing the forms.

After collection, all paper questionnaires were double-entered into a secure database by trained personnel to minimize errors. Quality control measures included checking for missing or inconsistent data, and verifying discrepancies with participants when possible.

All research staff involved in data collection received specialized training in data collection, ethical standards, and the use of questionnaire tools. Research assistants had a background in public health and were experienced in working with older adults. The lead researchers possessed extensive experience in community health research and intervention practices.

Inclusion Criteria: (1) Community residents aged 60 years and older, who are basically self-reliant in their daily lives, and have lived in the survey area for 1 year or more. (2) Individuals capable of normal language communication. (3) Informed consent and voluntary participation in this research.

Exclusion Criteria: (1) Older adults with disabilities, severe illnesses, or mental disorders who are unable to complete the survey. (2) Individuals or their family members who are unwilling to cooperate.

This study utilizes prior data mining analyses to estimate the community health service demands of older adults. Information on the basic characteristics and lifestyle behaviors of older adults is gathered through questionnaires. Scale scores were analyzed to assess the severity of health issues across various dimensions. Finally, statistical methods are applied to identify factors influencing the health of older adults.

### Statistical analysis

Statistical analysis was conducted using SPSS 24.0. Initially, basic descriptive analysis was performed. Normality tests were conducted for both categorical and continuous data, followed by chi-square tests for categorical variables where appropriate. For normally distributed data, *t*-tests, analysis of variance (ANOVA), and Pearson correlation tests were used. For non-normally distributed data, non-parametric tests were applied.

In a multi-factor analysis, as the dependent variable was binary categorical data, we employed stratified binary logistic regression with a significance level of *α* = 0.05 for hypothesis testing to investigate the factors influencing the health status of older adults.

### The selection of the scale

The assessment of older adults was conducted using the KCL (Katz Comorbidity List) scale, which is utilized to identify early signs of frailty and care requirements among older adults, thereby facilitating the provision of appropriate care services (9, 10). This scale comprises 25 items covering social activities, physical functioning, cognitive function, depressive emotions, and activities of daily living. It provides a comprehensive evaluation of older adults’ physical functioning, with two response options for each item: 0 for the ability to complete and 1 for the inability to complete. Frailty criteria include: a score of ≥10 on items 1–20, a score of 2 on items 11–12, a score of ≥3 on items 6–10, or a score of ≥2 on items 13–15. Meeting any of these criteria classifies an individual as frail; otherwise, they are classified as non-frail.

In this study, the total Cronbach’s *α* coefficient for the frailty scale was 0.916, indicating high reliability. Exploratory factor analysis yielded significant results (*p* = 0.000), with a Kaiser–Meyer–Olkin (KMO) measure of 0.931 and factor loadings above 0.4 for all dimensions, demonstrating that the scale has a robust structure and strong validity.

The assessment of psychological wellbeing was conducted using the General Health Questionnaire-12 (GHQ-12). This questionnaire, developed by David Goldberg in 1972 (11), is widely used for screening the psychological health of community populations and has received validation from various sources for its reliability and validity (12, 13). The GHQ-12 consists of 12 items, with four response options for each item. Scoring is done using a binary method (0-0-1-1), which means that for each item, selecting the first two response options scores 0, while choosing the last two response options scores 1. The final questionnaire score falls between 0 and 12, with higher scores indicating more severe psychological health issues.

Following the scoring standards of the World Health Organization, in this study, a cut-off value of 3 points was used. A total score of ≥3 indicates poor mental health in an individual, while a total score of <3 indicates psychological health.

In this study, the health status of older adults is comprised of two dimensions: physical frailty and psychological health, measured using the KCL frailty scale and the GHQ-12 questionnaire, respectively. Scores for both measures were standardized in the same direction, and both indicators are binary variables. They were combined to create a new variable called ‘comprehensive older adults health,’ with scores ranging from 0 to 2, where higher scores indicate poorer health. For reclassification, scores of ≥1 were categorized as ‘unhealthy’ (coded as 1), while scores <1 were categorized as ‘healthy’ (coded as 0).

## Results

Table 1 presents the basic demographic profile of community-dwelling older adults. The majority are female (56.7%) with an average age of 67.6 years. Over half have a normal weight (58.2%) and a monthly income above 3,000 yuan (71.5%). Most are married (84.0%) and live with others (86.0%). More than half have an education level of junior high school or below (60.8%). Nearly all older adults (97.1%) have spouses or children as caregivers. The vast majority are capable of self-care (96.0%) and do not experience anxiety (95.1%). Over 60% have chronic diseases (67.6%), and more than half rate their health as good (55.1%).

**Table 1:** Basic characteristics of participants [*n* (%)].

Variable	Divide into groups	Frequency (*n*)	Constituent ratio (%)
Sex	Man	575	43.3
	Woman	757	56.7
Age (years)	60–69	911	68.9
	70–79	365	27.6
	≥80	46	3.5
BMI	<18.5	29	2.2
	18.5 ≤ BMI ≤ 24.0	769	58.2
	≥25.0	524	39.6
Marital status	Married (with a spouse)	1,110	84.0
	Divorced or widowed/unmarried persons	212	16.0
Living situation	Living alone	185	14.0
	Not living alone	1,137	86.0
Monthly income (YUAN)	Below 1,000 yuan	61	4.6
	1,000–3,000 yuan	316	23.9
	More than 3,000 yuan	945	71.5
Degree of education	Junior high school and below	804	60.8
	High school/technical secondary school	377	28.5
	College degree or above	141	10.7
Types of caregivers	Spouses, children	1,284	97.1
	Non-immediate family members	9	0.7
	Take care of oneself	29	2.2
Take care of yourself	Complete self-help	1,269	96.0
	A little bit difficult	53	4.0
Anxious	No anxiety	1,257	95.1
	Have anxiety	65	4.9
Chronic disease	Not have	428	32.4
	Have	894	67.6

The three most prevalent chronic diseases are hypertension (48.63%), diabetes (24.43%), and coronary heart disease (6.79%). When categorized by gender, prevalence rates are as follows: hypertension (males 48.91%, females 48.39%), diabetes (males 25.96%, females 23.14%), and coronary heart disease (males 7.20%, females 6.45%).

The top three health maintenance methods are physical exercise (52.79%), maintaining a healthy lifestyle (18.59%), and controlling diet (13.99%).

A majority of older adults do not participate in social activities (76.0%), with only a small proportion frequently engaging in them (8.4%). Most undergo annual medical check-ups (68.1%), while a minority have never done so (6.7%).

In general, the most prevalent unhealthy lifestyle behaviors, ranked from highest to lowest, are poor sleep quality (88.4%), lack of attention to nutritional balance (32.3%), lack of personal interests (26.0%), lack of physical exercise (25.0%), smoking (15.9%), alcohol consumption (15.1%), and skipping breakfast (9.2%).

Gender analysis reveals that a higher proportion of females do not engage in physical exercise (27.3%) compared to males (22.0%) (*p* < 0.05). Males exhibit significantly higher rates of smoking (34.3%) and alcohol consumption (32.2%) compared to females (1.9 and 2.1%, respectively) (*p* < 0.001). No statistically significant differences between genders were observed for the other four indicators, as shown in Table 2.

**Table 2:** Basic living behaviors of older adults people in different gender communities [*n* (%)].

Life behavior		Male (*n* = 572)	Female (*n* = 750)	Total	χ^2^/*p*
Smoke	Yes	196 (34.3%)	14 (1.9%)	210 (15.9%)	χ^2^ = 254.935 *p* < 0.001
	No	376 (65.7%)	736 (98.1%)	1,112 (84.1%)	
Drink	Yes	184 (32.2%)	16 (2.1%)	200 (15.1%)	χ^2^ = 227.985 *p* < 0.001
	No	388 (67.8%)	734 (97.9%)	1,122 (84.9%)	
Movement	Yes	446 (78.0%)	545 (72.7%)	991 (75.0%)	χ^2^ = 4.867 *p* = 0.027
	No	126 (22.0%)	205 (27.3%)	331 (25.0%)	
Eat breakfast on time	No	52 (9.1%)	70 (9.3%)	122 (9.2%)	χ^2^ = 0.023 *p* = 0.880
	Yes	520 (90.9%)	680 (90.7%)	1,200 (90.8%)	
Sleep	Good	64 (11.2%)	90 (12.0%)	154 (11.6%)	χ^2^ = 0.027 *p* = 0.649
	Bad	508 (88.8%)	660 (88.0%)	1,168 (88.4%)	
Personal interest	Have	416 (72.7%)	562 (74.9%)	978 (74.0%)	χ^2^ = 0.820 *p* = 0.365
	Not have	156 (27.3%)	188 (25.1%)	344 (26.0%)	
Nutritive equilibrium	Yes	388 (67.8%)	507 (67.6%)	895 (67.7%)	χ^2^ = 0.008 *p* = 0.929
	No	184 (32.2%)	243 (32.4%)	427 (32.3%)	

The psychological wellbeing of older adults in Dalian’s community was assessed using the General Health Questionnaire (GHQ-12). The proportion of psychologically unhealthy older adults was 2.87% (38/1322). According to the Frailty KCL scale, the prevalence of frailty among community-dwelling older adults was 3.10% (41/1,322). Early signs of frailty primarily manifested as declines in lower limb muscle function and memory, with declines in physical and cognitive function being relatively common.

The total score for older residents in the community was 349 points. Regarding psychological health, the severity of issues from highest to lowest was: anxiety > impaired social functioning > loss of self-confidence. For frailty, the 25 items were categorized into seven major dimensions reflecting physical, psychological, mental, and social activities. The severity of frailty from highest to lowest in each dimension was: activities of daily living > physical function > cognitive function > nutritional status > oral cavity function > social activities > depressive emotions, as shown in Table 3.

**Table 3:** Scores in various dimensions of psychological wellbeing and frailty status among older adults (*n*).

Category	Dimension	Clauses and subclauses	Score	Dimension total score	Dimension average score
Psychological wellbeing	Anxious	3. Mental stress	69	198	49.5
		4. Lack of willpower	26		
		5. Low mood	30		
		8. Worry and insomnia	73		
	Impaired social functioning	1. A waste of time	27	125	20.8
		2. Do not hesitate	29		
		6. Do not enjoy life	17		
		7. Escape from difficulties	16		
		10. Inconcentration	19		
		12. Not happy	17		
	Loss of confidence	9. Loss of confidence	13	26	13.0
		11. No value	13		
Frailty status	Activities of daily living	1. Go out independently	21	881	176.2
		2. Buy items	20		
		3. Deposit and withdrawal	24		
		4. Visit chat	791		
		5. Discuss with your family	25		
	Physical function	6. Ascend the stairs	38	261	52.2
		7. Stand	26		
		8. 15 min on foot	17		
		9. Fell down within 1 year	73		
		10. Fear of falling down	107		
	Cognitive function	17. Memory deterioration	91	134	44.7
		18. Make an independent call	14		
		19. Date	29		
	Nutritional status	11. Loss of weight	42	42	42
	Oral cavity function	12. Chewing ability	30	125	41.7
		13. Bucking	36		
		14. Thirsty	59		
	Social activities	15. Leave home	17	46	23
		16. The number of out	29		
	Depressive emotions	20. Life is boring	23	105	21
		21. Vapidity	22		
		22. Too lazy to do	22		
		23. Feeling of uselessness	16		
		24. Tired	22		

The average score of the dimension is the number of items in the dimension and divided by the number of items in the dimension.

In the overall population of older adults, the detection rate for overall health was 84.40%, while the rate for unhealthy individuals was 15.60%.

Based on the health ecology model and guided by literature review findings, independent variables were selected. Factors related to living and working conditions and policy aspects were challenging to identify, so theoretical model frameworks were used to analyze these factors. The evaluation of older adults’ health focused on three levels: personal characteristics, lifestyle behaviors, and social networks. Personal characteristics included gender, age group, BMI, monthly income, educational level, chronic diseases, self-care ability, anxiety, and self-rated health. Lifestyle behaviors included smoking, alcohol consumption, exercise, personal interests, sleep, regular breakfast consumption, and nutritional balance. Social network factors included marital status, living situation, and caregiver type. Each categorical variable was transformed into dummy variables, with one category serving as the reference (the first category for each variable).

Chi-square analysis was conducted using the comprehensive health assessment results as the dependent variable. Since most variables were categorical, with a few continuous variables converted into categories, the Chi-square test was employed, as shown in Table 4, which presents the factors related to the health of older adults.

**Table 4:** Analysis of factors affecting health in older adults.

Administrative levels	Variable				
Individual trait	Monthly income	Degree of education	Chronic disease	Take care of yourself	Anxious
	12.14**	5.28*	15.62**	31.87**	67.63**
	Health self-evaluation				
	29.90**				
Life behavior	Movement	Personal interest	Sleep	Eat breakfast on time	Nutritive equilibrium
	17.10**	3.90*	16.46**	10.06**	30.54**

*p < 0.05, **p < 0.001.

Using the comprehensive health assessment results for older adults (0 = healthy, 1 = unhealthy) as the dependent variable, significant variables from the correlation analysis were chosen as independent variables. Based on the health ecology model, variables related to the health status of community-dwelling older adults were gradually included in a stratified regression analysis. Monthly income, chronic diseases, self-care ability, anxiety, self-rated health, and other personal traits were included as the first layer of independent variables in the initial regression. In the second step, lifestyle behaviors such as smoking, alcohol consumption, exercise, sleep, and breakfast were added as the second layer to examine the factors influencing health.

Binary logistic regression was conducted with a significance level of *α* = 0.05. The data met the requirements for constructing a binary logistic regression model, with no multicollinearity observed (all variance inflation factors (VIF) < 10). Therefore, the data were deemed suitable for building the stratified model.

The multivariate analysis results are presented in Table 5. Hosmer–Lemeshow test *p*-values were all greater than 0.1, indicating a good model fit. By analyzing the *R*^2^ values for each layer, it was possible to infer the impact of each independent variable. When variables from personal traits and lifestyle behaviors were sequentially added, *R*^2^ values were statistically significant. Model II showed a better fit compared to Model I and was analyzed further.

**Table 5:** Binary stratified logistic regression for health in community older adults.

Administrative levels	Variable (reference group)	Model I	Model II
		OR value (95% CI)	OR value (95% CI)
Individual trait	Chronic disease (none = 0)		
	Have	2.777** (1.288–5.986)	2.629* (1.192–5.795)
	Self-care of life (can = 0)		
	Difficulty	3.699** (1.732–7.901)	3.807** (1.628–8.904)
	Anxiety (none = 0)		
	Have	4.758** (2.476–9.145)	4.377*** (2.119–9.039)
	Self-rated health (good = 0)		
	Mal	2.414** (1.415–4.119)	1.912* (1.090–3.353)
Life behavior	Sleep (good = 0)		
	Mal		1.990* (0.991–3.993)
	Nutritional balance (Yes = 0)		
	No		1.183^*^ (0.795–3.055)
	−2 Log-likelihood value	493.908	440.172^a^
	Cox-Snell R^2^	0.056	0.094
	Nagelkerke R^2^	0.160	0.268
	Coefficient comprehensive test	*p* < 0.01	*p* < 0.01
The Hosmer–Lemeshow test			
	Chi-square value	2.103	7.788
	Free degree	4	8
	P price	0.717	0.454

*p < 0.05, **p < 0.01, ***p < 0.001.

Older adults with chronic diseases had a 2.629 times higher risk of being unhealthy compared to those without chronic diseases. Those with difficulties in self-care had a 3.807 times higher risk, and those with anxiety had a 4.377 times higher risk. Poor self-rated health was associated with a 1.912 times higher risk, poor sleep quality with a 1.99 times higher risk, and nutritional imbalances with a 1.183 times higher risk.

The regression results indicate that chronic diseases, self-care ability, anxiety, self-rated health, sleep, and nutritional balance are significant factors affecting the health of community-dwelling older adults. Chronic diseases, inability to manage self-care, anxiety, poor self-rated health, poor sleep quality, and nutritional imbalance are all risk factors for poor health in this population.

The community under investigation is managed by community health service organizations responsible for older adults’ health management. The provided services align with the “National Basic Public Health Service Standards (Third Edition)” and primarily include health check-ups, health record establishment, and health education. Community health organizations offer annual check-ups for individuals aged 65 and above, which include routine physical examinations and auxiliary tests. Physical examinations cover height, weight, and blood pressure, while auxiliary tests include electrocardiograms, ultrasounds, and blood tests. Community doctors provide lifestyle and medication guidance to older adults with abnormal results through interviews and examination reports and compile the collected information to establish health records.

Health education for older adults involves inviting experts from tertiary hospitals to conduct regular health lectures and clinics at the grassroots level. Residents receive health booklets and watch health promotion videos as part of these activities. The mode of health management services remains traditional, with older adults visiting community health centers independently to receive care.

## Discussion

This study examines the physical and mental health status and influencing factors of older adults living in communities in Dalian, Northeast China. Using cross-sectional data, it assesses the prevalence of chronic diseases, frailty, and psychological issues such as anxiety, while identifying key risk factors affecting their health.

The survey results indicate that in Dalian, 3.10% of older adults are either in the early stages of frailty or have already exhibited signs of frailty. A longitudinal cohort study showed that the age-adjusted prevalence of frailty among community-dwelling older adults ranged from 2.3% in the southeast and northeast to 9.1% in the northwest (14). The average scores for various dimensions of frailty reveal that the decline in daily life activities and physical functions is the most severe among older adults. Both stages of the survey identified that early signs of frailty in older adults primarily manifest in the decline of muscle function in the lower limbs and a reduction in the frequency of social outings. This finding aligns with research conducted by Cooper (15). The results indicated an increased likelihood of muscle weakness (difficulty climbing stairs) among older adults. Those who used community health services to enhance physical activity before the pandemic may have reduced their exercise during the pandemic due to control measures (16). These measures also led to a decrease in social interaction among relatively healthy older adults, which increased the risk of physical frailty. The decrease in outings not only brings the risk of reduced lower limb skeletal muscle leading to disability but also lowers social engagement, thereby posing a risk of older adults depression (17–19). In the post-pandemic era, older adults should gradually increase their outings if capable. Communities should engage in effective outreach and organize activities specifically designed to improve lower limb function, thereby encouraging more frequent outings among older adults.

The proportion of older adults in Dalian experiencing psychological distress is 2.87%. Results across different dimensions of psychological wellbeing indicate that anxiety and impaired social functioning are the most severe issues among older adults. Psychological distress in older adults primarily manifests as mental stress and concerns that lead to insomnia. Relevant research has shown that during the pandemic, anxiety and depression were common psychological problems, likely associated with pandemic control measures (20). The reduced frequency of social outings among older adults, as mentioned earlier, may have contributed to these results. Older adults with lower social interaction are at higher risk of experiencing anxiety (21). Older adults are encouraged to engage in social activities and group events, which can help reduce the risk of psychological distress.

The regression results indicate that chronic illnesses, self-care ability, anxiety, self-assessment of health, sleep, and nutritional balance are significant factors influencing the health of older adults in the community. This is in concordance with relevant studies (22). Notably, comorbidity of chronic illnesses in older adults leads to various severe lifelong physical symptoms (23), impacting anxiety and depression among community-dwelling older adults. Additionally, it has a pronounced effect on both their physical and psychological health. Other personal characteristics among older adults directly reflect their physical and psychological health status, thus exerting an influence on their overall wellbeing.

Sleep quality significantly affects the physical and psychological health of older adults and is closely linked to frailty (24). This relationship is exacerbated when older adults spend prolonged periods at home, leading to disrupted and fragmented daytime sleep. Research indicates that improving sleep quality is an effective strategy for preventing and delaying frailty. In the post-pandemic era, reducing daytime home confinement is advisable for older adults. This can be achieved by enhancing lower limb function through exercise and minimizing fragmented daytime sleep to slow frailty progression.

The physical and mental health challenges faced by older adults during the pandemic varied between China and other countries. The prevalence of chronic diseases, especially hypertension and diabetes, is high among older adults in China (25). These chronic diseases significantly impact the quality of life and self-care abilities of older adults, heightening their anxiety and vulnerability. In China, pandemic prevention measures reduced social activities and outings for older adults, resulting in declines in physical function and increased psychological stress. In contrast, older adults in some other countries maintained social contact through social media and digital health services (26). Regional and cultural differences must be considered when developing health management strategies for older adults. Community health interventions in China should prioritize improving chronic disease management and promoting social engagement among older adults to better meet their needs.

This study holds significant practical implications for community health management. The results indicate the need for targeted interventions, focusing on increasing physical activities, promoting social interaction, and managing chronic diseases among older adults. Community-based programs that promote exercise, improve nutrition, and alleviate anxiety can help reduce frailty and psychological distress in older adults. The findings can serve as a reference for policymakers, helping them adjust the provision of health services based on the specific needs of older adults.

Implementing specific community-based interventions, such as mental health support programs, nutritional education, and social and physical activities, can mitigate health risk factors like anxiety and nutritional imbalance among older adults. Communities can organize regular mental health lectures and support groups to help older adults manage emotions and reduce feelings of loneliness; promote nutritional awareness by offering personalized dietary guidance to ensure adequate nutrient intake; and provide tailored recreational and exercise programs that increase physical activity levels and social engagement. These interventions provide practical guidance for fostering healthy aging and enhancing the overall wellbeing of older adults within the community.

Future research should investigate the long-term impact of the COVID-19 pandemic on both the physical and mental health of older adults. Additionally, the role of digital health interventions, such as remote monitoring and telehealth services, in supporting older adults’ health warrants further investigation. More comprehensive randomized studies are needed to confirm these findings and to evaluate the effectiveness of targeted community health interventions in improving the health of older adults. Furthermore, researchers should consider cross-regional comparisons of health outcomes to identify best practices for older adult care.

## Conclusion

This study highlights the physical and mental health status of older adults in Dalian, Northeast China, identifying chronic diseases, frailty, and anxiety as prevalent issues. Among chronic diseases, hypertension, diabetes, and coronary heart disease are the most common. Older adults primarily choose physical exercise, maintaining a healthy lifestyle, and dietary control as self-care strategies. Early signs of frailty are mainly characterized by declines in lower limb muscle function and memory. Anxiety is also a common indicator of poor health in this population. Key risk factors for older adults’ health include chronic diseases, impaired ability to perform daily activities, anxiety, poor self-rated health, sleep disturbances, and nutritional imbalance. The findings suggest that targeted community interventions focusing on physical activity, social engagement, and chronic disease management are essential to improving the health and wellbeing of older adults. These insights provide valuable guidance for policymakers and healthcare providers to tailor health services to meet the specific needs of this population.

### Strengths

This study adopts an early health intervention perspective and establishes a research framework based on the Health Ecology model and community health intervention theories. Field investigations and questionnaire surveys were conducted over 2 years to analyze the health status and influencing factors of the older adult population in Dalian. Data analysis provided insights into the current state of community health management for older adults. The methodology is advanced, reliable, and grounded in well-established theory.

### Limitations

This study was conducted from 2020 to 2022. Due to the COVID-19 pandemic, conducting door-to-door surveys and data collection was challenging, making random sampling impractical. Instead, questionnaires were distributed and data were collected by community healthcare personnel using a convenience sampling method. This approach, while practical under the circumstances, may affect the representativeness of the sample, limiting the generalizability of the results. The data are considered authentic and reliable, reflecting the health status of older adults in the study area.

However, the study has several limitations. First, convenience sampling may result in a lack of sample representativeness. Second, data collection during the pandemic, coupled with control measures, may have temporarily impacted older adults’ health. Third, the cross-sectional design prevents establishing causal relationships; thus, future studies should adopt longitudinal approaches. Additionally, self-reported data are prone to recall and social desirability biases; combining objective health assessments is recommended to improve reliability. Finally, the assessment tools may not fully capture the overall health status of older adults, and future research should consider multidimensional evaluations.

## Data Availability

The original contributions presented in the study are included in the article/Supplementary material, further inquiries can be directed to the corresponding authors.
